# Facial palsy during the COVID‐19 pandemic

**DOI:** 10.1002/brb3.1939

**Published:** 2020-11-07

**Authors:** Luca Codeluppi, Francesco Venturelli, Jessica Rossi, Antonio Fasano, Giulia Toschi, Francesca Pacillo, Francesco Cavallieri, Paolo Giorgi Rossi, Franco Valzania

**Affiliations:** ^1^ Neurology Unit Neuromotor & Rehabilitation Department Azienda USL‐IRCCS di Reggio Emilia Reggio Emilia Italy; ^2^ Clinical and Experimental Medicine PhD Program University of Modena and Reggio Emilia Modena Italy; ^3^ Epidemiology Unit Azienda USL‐IRCCS di Reggio Emilia Reggio Emilia Italy; ^4^ Department of Biomedical, Metabolic and Neural Sciences Center for Neuroscience and Neurotechnology University of Modena and Reggio Emilia Modena Italy; ^5^ Neurology Department OCB Hospital AOU Modena Modena Italy

**Keywords:** Bell's palsy, COVID‐19, facial palsy, nerve, SARS‐CoV‐2

## Abstract

**Objective:**

To compare the incidence and clinical features of individuals presenting in emergency rooms (ER) with facial palsy during the Italian COVID‐19 outbreak and in the same period of 2019.

**Methods:**

We retrospectively reviewed the medical records for all accesses to the six ER in the province of Reggio Emilia, Italy, during the first phase of the COVID‐19 pandemic (27 February‐3 May 2020) to identify all cases of diagnosed facial palsy. Clinical information was retrieved for each patient and compared with that of facial palsy cases presenting in 2019.

**Result:**

Between 27 February and 3 May 2020, 38 patients presented to provincial ERs for facial palsy; in 2019, there were 22 cases, for an incidence rate ratio of 1.73 (95% CI 1.02–2.92) for the 2020 cohort. Of the 2020 cohort, eight patients (21%) presented with active or recent symptoms consistent with COVID‐19 infection, compared with 2 (9%) in 2019 (*p* = .299); one was tested and resulted positive for *SARS‐CoV‐2*. Moreover, patients were younger (−11 years, *p* = .037) than those of the previous year and manifested a longer lag (+1.1 days, *p* = .001) between symptoms onset and ER presentation.

**Conclusion:**

We observed a higher occurrence of facial palsy during the COVID‐19 outbreak compared to the same period of the previous year; 21% of patients presenting with facial palsy had active or recent symptoms consistent with *SARS‐CoV‐2* infection, suggesting an excess risk of facial palsy during or after COVID‐19. These patients searched for medical attention later, probably because of the fear of contracting COVID‐19 during assistance.

## Graphical abstract

The objective of this study is to describe the clinical features of individuals presenting with facial palsy during the Italian COVID‐19 outbreak. We retrospectively reviewed the medical records for all accesses to the emergency departments in the province of Reggio Emilia, Italy, during the first phase of the COVID‐19 pandemic (27 February‐3 May 2020) to identify all cases of diagnosed facial palsy. We observed a higher occurrence of facial palsy during the COVID‐19 outbreak compared to the same period of the previous year; 21% of patients presenting with facial palsy had active or recent symptoms consistent with SARS‐CoV‐2 infection, suggesting a potential correlation.

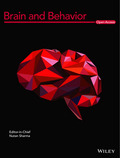

## 1 INTRODUCTION

The SARS‐CoV‐2 infection (COVID‐19) (Zhu et al., 2020) pandemic continues to disrupt daily life and is still a major public health issue worldwide in 2020. Italy has been greatly affected, and recent data show that 2.5% of healthy adults could have evidence of *SARS‐CoV‐2* antibodies by the end of May, with seroprevalence up to 24% in some northern areas. (ISTAT Primi risultati dell’indagine di sieroprevalenza sul SARS‐CoV‐2, 2020) During the initial phase of the spread of the virus, the Italian government imposed tight restrictions on mobility (lockdown) between 8 March and 3 May, the period during which the incidence of COVID‐19 cases reached its peak. The province of Reggio Emilia, in northern Italy (population 530,000 inhabitants) (https://statistica.regione.emilia‐romagna.it/servizi‐online/statistica‐self‐service/popolazione/popolazione‐per‐eta‐e‐sesso/pop_eta_ammontare), has had high rates of infection and death: 4,765 subjects (0.90%) had tested positive for *SARS‐CoV‐2* on nasopharyngeal swabs (https://statistica.regione.emilia‐romagna.it/servizi‐online/statistica‐self‐service/popolazione/popolazione‐per‐eta‐e‐sesso/pop_eta_ammontare) by the end of lockdown on 3 May. In March 2020 alone, there were 224 deaths due to COVID‐19 (https://www.istat.it/it/files//2020/05/Rapporto_Istat_ISS.pdf.). Considering that many subjects (especially if asymptomatic or paucisymptomatic) were not tested due to health system overload, the real number of infected patients is likely to be higher. *SARS‐CoV‐2* infection is typically marked by fever and respiratory symptoms (Guan et al., 2020), although neurologic manifestations of the disease have also been described (Vonck et al., 2020). Recent studies have reported that *SARS‐CoV‐2*‐related infection may present with cranial neuropathies (Dinkin et al., 2020; Goh et al., 2020), and facial nerve palsy in *SARS‐CoV‐2*‐positive patients has so far been described both as isolated and unilateral (Goh et al., 2020) or bilateral in the context of Guillain–Barre syndrome (Helbok et al., 2020). The mechanisms by which *SARS‐CoV‐2* may cause cranial neuropathies have recently been hypothesized (Costello & Dalakas, 2020).

Idiopathic facial palsy (Bell's palsy) is a common cranial mononeuropathy (Eviston et al., 2015) routinely seen in clinical practice whose cornerstone treatment is based mainly on a short course of oral corticosteroids (Madhok et al., 2016) and antiherpetic drugs (Eviston et al., 2015). The cause of facial palsy is still unclear although a correlation with viral infection has been claimed (McCormick, 1972; Peitersen, 2002). Moreover, the increased incidence of facial palsy observed during a vaccine trial has suggested that an immune‐mediated mechanism must be considered (Lewis et al., 2009). *SARS‐CoV‐2* damage is thought to be caused also by immunity activation (Costello & Dalakas, 2020), and coronaviruses are known to be neurotropic (Natoli et al., 2020), so there is the basis to consider a link between COVID‐19 and facial palsy.

The aim of our study was to analyze the variation in the incidence and clinical features of patients presenting with facial palsy during the early and lockdown phases of the COVID‐19 pandemic in our province to determine whether there are any potential links between the two conditions.

## 2 MATERIALS AND METHODS

All the emergency departments in the province of Reggio Emilia are under the Local Health Authority (LHA) (Azienda Sanitaria Locale‐IRCCS di Reggio Emilia), which has single data warehouse that shares data between the hub hospital and the five spoke hospitals. All neurological visits for acute conditions are performed through the LHA and are trackable.

We retrospectively reviewed the electronic medical records for all accesses to our LHA from 27 February 2020, when the first confirmed COVID‐19 patient in our province was registered, to 3 May, when lockdown ended, to identify all cases of idiopathic facial palsy.

For each patient, we gathered data on age, sex, presence of diabetes, pregnancy, days from symptom onset to seeking medical assistance, and whether corticosteroids had been prescribed. We also searched clinical reports for active or recent symptoms consistent with COVID‐19 infection (considering recent as less than 14 days from facial palsy onset), test results for *SARS‐CoV‐2* infection (when performed) and subsequent accesses to the LHA in the 30 days following facial palsy. We considered consistent with COVID‐19 all symptoms ascribable to *SARS‐CoV‐2* infection as listed by the World Health Organization on May 2020 (fever, dyspnea, cough or other respiratory symptoms, fatigue, headache, myalgia, diarrhea, nausea or vomiting, alteration of taste or smell) (https://www.who.int).

Occurrences and characteristics of cases during this initial phase of the pandemic were compared with those of patients observed in the same period in the previous year (26 February – 3 May 2019, backdating the period by adding one day to compensate for the leap year in 2020). All patients diagnosed with facial palsy were identified, and the same basic features with the same criteria were collected; we also searched medical records for symptoms consistent with recent or active infection of any type at presentation. We used the resident population of the province of Reggio Emilia on 1 January 2019 and 1 January 2020 (https://statistica.regione.emilia‐romagna.it/servizi‐online/statistica‐self‐service/popolazione/popolazione‐per‐eta‐e‐sesso/pop_eta_ammontare) to calculate the incidence rate of facial palsy per 100,000 people during the period under study during the pandemic and in the same calendar period in 2019. The incidence rates were then compared calculating the risk ratio (RR) with relative 95% confidence intervals (95% CI). Statistical analysis was performed using the IBM SPSS Statistics for Windows version 20.0 (IBM). Continuous variables were expressed as mean (±*SD*) and median (range), while frequencies and percentages were calculated for categorical variables. Because continuous variables were not normally distributed (Kolmogorov–Smirnov test), we applied the U‐statistic to compare the two groups. Mean values were also compared by using the Student's t‐distribution. We compared the frequencies of the remaining variables in the 2019 and 2020 groups by applying the Fisher exact distribution. The study was approved by the Area Vasta Emilia Nord Ethics Committee on 9 June 2020 (protocol number 2020/0045199).

## 3 RESULTS

Thirty‐eight patients accessed the local emergency departments for facial palsy during the period in 2020 under study (incidence rate 7.1 cases per 100,000 inhabitants), while only 22 patients were found in the same calendar period of 2019 using the same medical information sources (incidence rate 4.1 cases per 100,000 inhabitants), resulting in a significantly higher incidence in 2020 compared to 2019 (RR 1.73; 95% CI 1.02–2.92). The main features of the two groups are summarized in Table 1. All patients included had data for the variables in the study.

**TABLE 1:** Patients' characteristics

Variable	2019 cohort Total *n* = 22	2020 cohort Total *n* = 38	*p*‐value for equal population in 2020 and 2019
Sex; No. (%)			
Male	11 (50%)	22 (57.9%)	.599^$^
Female	11 (50%)	16 (42.1%)	
Age			
No. (%), mean, [±*SD*];	64.95 [±19.52];	53.53 [±20.19];	.037^§^
Median {range; interquartile range}	66.50 {18–90; 24}	55.50 {15–84; 30}	.026*
Presence of active or recent infectious symptoms; No. (%)			
No	20 (90.90%)	30 (78.90%)	.299^$^
Yes	2 (9.10%)	8 (21.10%)	
Days from onset to seeking medical attention			
No. (%), mean, [±*SD*];	0.55 [±0.51];	1.68 [±1.64];	.003^§^
Median, {range; interquartile range}	1.00 {0–1; 1}	1.00 {0–7; 1}	.001*
Diabetes; No. (%)			
No	15 (68.20%)	30 (78.90%)	.372^$^
Yes	7 (31.80%)	8 (21.10%)	
Autoimmune diseases; No. (%)			
No	21 (95.50%)	35 (92.10%)	1.000^$^
Yes	1 (4.50%)	3 (7.90%)	
Immunosuppressive therapy; No. (%)			
No	21 (95.50%)	37 (97.40%)	1.000^$^
Yes	1 (4.50%)	1 (2.60%)	
Prescription for steroid treatment; No. (%)			
No	3 (13.60%)	7 (18.40%)	.732^$^
Yes	19 (86.40%)	31 (81.60%)	

^$^ *p*‐value for Fisher exact distribution.

^§^ *p*‐value for Student's t‐distribution.

* i‐value for U statistic.

Patients presenting at an ED during the 2020 period were younger than those presenting in 2019 (median age 55.50 versus. 66.50, *p* = .026). In 2020, 8 out of 38 patients (21%) had active or recent infectious symptoms at the time of presentation, compared with two out of 22 (9.10%) in 2019 (*p* = .299). Moreover, the lag between the onset of the facial palsy and the seeking of medical attention was longer in the 2020 group (median = 1.68 wdays, *n* = 38) than in the 2019 group (median = 0.55 days, *n* = 22), p for equal median* *= .001. We did not find any difference between the rate of diabetes, previous autoimmune disease, or active immunosuppressive therapy. Pregnancy rate was unremarkable, as only one patient was pregnant in 2019 and none in 2020. The number of patients who were prescribed corticosteroid therapy by a neurologist was similar in the two periods. Due to the worsening of symptoms, one patient in the 2020 group was admitted to a respiratory care unit and was tested for *SARS‐CoV‐2* on nasopharyngeal swab, which resulted positive. The remaining 37 patients were not tested for *SARS‐CoV‐2* due to absent or mild infectious symptoms and to the health system overload during this stage of the pandemic. Thirty‐one patients were prescribed steroid therapy to treat facial palsy (3 with active or recent symptoms of infection at presentation) and none subsequently represented to the LHA for the appearance or worsening of infectious symptoms.

## 4 DISCUSSION

We observed a higher occurrence of facial palsy in the pandemic period compared to the same period of the previous year. Moreover, about 21% of patients presenting to the emergency department for facial palsy during COVID‐19 outbreak had active or recent symptoms consistent with COVID‐19 infection. Although not definitive, these observations may support the association between acute mononeuropathies and COVID‐19, as recently described (Costello & Dalakas, 2020; Dinkin et al., 2020; Goh et al., 2020). Patients affected by facial palsy during the COVID‐19 pandemic have found also to be younger, even if we do not have enough elements so far to reasonably hypothesize the cause of this association.

While COVID‐19 is clearly a very serious disease on its own, it has also appears to have an indirect negative effect on other pathologies due to the impaired response of an overloaded health system (Driggin et al., 2020; Roberton et al., 2020). Further, we think the fear of contracting COVID‐19 may lead patients to forego or delay seeking medical attention. Supporting this hypothesis is the lag we found between disease onset and presentation in patients with facial palsy during the COVID‐19 outbreak. This has even more value when considering that facial palsy rarely goes unnoticed, patients usually are very alarmed by its symptoms and have little or no knowledge of the disease. Considering that patients’ fears during a pandemic may play a role in their delaying or even foregoing access to healthcare highlights the importance of information campaigns and territorial medical assistance. Our study has several limitations, the first being that only one of the patients was tested for *SARS‐CoV‐2*, primarily due to the health system overload during the acute phase of the pandemic, when nasopharyngeal swabs were performed only on severe cases. Moreover, we have evaluated only those patients visited by neurologists thereby potentially missing subjects managed exclusively by general practitioners.

In conclusion, the main result we observed was a higher incidence of facial palsy during the COVID‐19 pandemic. Although not enough to establish a clear causative relationship between the two conditions, these data may suggest that COVID‐19 could hide even behind common clinical conditions like facial palsy suggesting physicians to keep the alert high during standard clinical practice.

## CONFLICT OF INTEREST

All authors report no disclosures relevant to the manuscript.

## AUTHOR CONTRIBUTION

1. Research project: A. Conception, B. Organization, C. Execution; 2.Data Analysis: A. Design, B. Execution, C. Review/ Critique; 3. Manuscript Preparation: A. First draft, B. Review/Critique. LC conceived, organized, and executed research project; designed data analysis; and first drafted and reviewed/critiqued manuscript preparation. FV conceived and executed research project; designed and executed data analysis; and first drafted and reviewed/critiqued manuscript preparation. JR, AF, GT, and FP executed research project; executed data analysis; and reviewed/critiqued manuscript preparation. FC conceived, organized, and executed research project; designed and executed data analysis; and first drafted manuscript preparation. PGR and FV conceived, organized, and executed research project; designed and executed data analysis; and first drafted and reviewed/critiqued manuscript preparation.

## ETHICS COMMITTEE APPROVAL & PATIENT CONSENT

The protocol of the study was approved by the local Institutional Review Board. The study protocol was in accordance with the 1964 Helsinki Declaration and its later amendments comparable ethical standards.

### Peer Review

The peer review history for this article is available at https://publons.com/publon/10.1002/brb3.1939.

## Data Availability

The data that support the findings of this study are available from the corresponding author upon reasonable request.
